# Voltage during atrial fibrillation is superior to voltage during sinus rhythm in localizing areas of delayed enhancement on magnetic resonance imaging: An assessment of the posterior left atrium in patients with persistent atrial fibrillation

**DOI:** 10.1016/j.hrthm.2019.05.032

**Published:** 2019-09

**Authors:** Norman A. Qureshi, Steven J. Kim, Chris D. Cantwell, Valtino X. Afonso, Wenjia Bai, Rheeda L. Ali, Matt J. Shun-Shin, Louisa C. Malcolme-Lawes, Vishal Luther, Kevin M.W. Leong, Elaine Lim, Ian Wright, Szabi Nagy, Sajad Hayat, Fu Siong Ng, Michael Koa Wing, Nick W.F. Linton, David C. Lefroy, Zachary I. Whinnett, D. Wyn Davies, Prapa Kanagaratnam, Nicholas S. Peters, Phang Boon Lim

**Affiliations:** ∗ Imperial College Healthcare NHS Trust, Hammersmith Hospital, London, United Kingdom; † Abbott Medical Inc, St Paul, Minnesota; ‡ Imperial College London, London, United Kingdom

**Keywords:** Atrial fibrillation, Atrial fibrosis, Atrial fibrillation voltage, Magnetic resonance imaging

## Abstract

**Background:**

Bipolar electrogram voltage during sinus rhythm (V_SR_) has been used as a surrogate for atrial fibrosis in guiding catheter ablation of persistent atrial fibrillation (AF), but the fixed rate and wavefront characteristics present during sinus rhythm may not accurately reflect underlying functional vulnerabilities responsible for AF maintenance.

**Objective:**

The purpose of this study was determine whether, given adequate temporal sampling, the spatial distribution of mean AF voltage (V_mAF_) better correlates with delayed-enhancement magnetic resonance imaging (MRI-DE)–detected atrial fibrosis than V_SR_.

**Methods:**

AF was mapped (8 seconds) during index ablation for persistent AF (20 patients) using a 20-pole catheter (660 ± 28 points/map). After cardioversion, V_SR_ was mapped (557 ± 326 points/map). Electroanatomic and MRI-DE maps were co-registered in 14 patients.

**Results:**

The time course of V_mAF_ was assessed from 1–40 AF cycles (∼8 seconds) at 1113 locations. V_mAF_ stabilized with sampling >4 seconds (mean voltage error 0.05 mV). Paired point analysis of V_mAF_ from segments acquired 30 seconds apart (3667 sites; 15 patients) showed strong correlation (r = 0.95; *P* <.001). Delayed enhancement (DE) was assessed across the posterior left atrial (LA) wall, occupying 33% ± 13%. V_mAF_ distributions were (median [IQR]) 0.21 [0.14–0.35] mV in DE vs 0.52 [0.34–0.77] mV in non-DE regions. V_SR_ distributions were 1.34 [0.65–2.48] mV in DE vs 2.37 [1.27–3.97] mV in non-DE. V_mAF_ threshold of 0.35 mV yielded sensitivity of 75% and specificity of 79% in detecting MRI-DE compared with 63% and 67%, respectively, for V_SR_ (1.8-mV threshold)_._

**Conclusion:**

The correlation between low-voltage and posterior LA MRI-DE is significantly improved when acquired during AF vs sinus rhythm. With adequate sampling, mean AF voltage is a reproducible marker reflecting the functional response to the underlying persistent AF substrate.

## Introduction

Atrial fibrosis is known to play an important role in the maintenance of atrial fibrillation (AF). By interfering with electrical continuity, fibrotic tissue is vulnerable to slow conduction, refractory dispersion, and functional reentry, perpetuating AF.^1^ The optimal method of identifying *de novo* atrial fibrosis in persistent AF remains unclear. Delayed-enhancement magnetic resonance imaging (MRI-DE) has been used to visualize fibrotic remodeling^2^, ^3^, ^4^ but is limited by its current scale of resolution. More recently, low-amplitude bipolar electrograms (EGMs) have been used as an electrical surrogate for fibrosis while mapping during sinus rhythm (SR).^5^, ^6^ However, EGM amplitudes can be influenced by many factors. In addition to recording electrode characteristics (size, spacing, bipole orientation), physiological rate and wavefront dynamics have a direct bearing on the resultant EGM characteristics.^7^ Hence, EGM amplitudes associated with fixed, directional conduction during SR can be misleading, presenting a major challenge to discrimination of the fibrotic substrate. In contrast, AF presents as a variable-rate and multidirectional rhythm. Being the clinical rhythm of interest, we hypothesized that AF provides the ideal paradigm for substrate interrogation in order to better resolve the underlying functional vulnerabilities most relevant to AF perpetuation. Given conditions of adequate sampling, EGM voltage measured during AF should be a reproducible marker of the underlying substrate, allowing for a more sensitive and specific measure of MRI-DE detected fibrosis compared to voltage acquired during SR.

## Methods

### Study population

The study comprised 20 patients presenting for persistent AF ablation (Table 1). Local Ethics Committee approval was obtained.

**Table 1:** Patient clinical demographics, MRI, and mapping parameters of patients recruited into the study

Patient characteristics (n = 20)	
Age (y)	62 ± 11
Male	11 (55)
Mean LA size on TTE (mm)	41 ± 6
Mean CHA_2_DS_2_VASc score	2.4 (0–6)
Hypertension	8 (40)
Diabetes mellitus	4 (20)
Cerebrovascular disease	2 (10)
History of heart failure	4 (20)
Duration of persistent AF (mo)	21.3 ± 10

MRI and electrophysiologic parameters (n = 14)∗		
MRI surface area	DE†	Non-DE
Left atrium (cm^2^) (%)	37.6 ± 9.4 (27.0 ± 9.8)	108.6±30.4 (73.0 ± 9.8)
Posterior LA (cm^2^) (%)	20.1 ± 9.3 (33.4 ± 13.2)	40.5±14.0 (66.6 ± 13.2)

Rhythm (n)	AF‡ (14)	Sinus rhythm (13)
Map points (density)		
Left atrium (pts/cm^2^)	660 ± 283 (4.6 ± 1.9)	527±326 (4.0 ± 2.8)
Posterior LA (pts/cm^2^)	306 ± 109 (5.0 ± 1.3)	284±103 (4.9 ± 1.9)
Voltage (mV)§		
Global	0.35 [0.19–0.61]	1.81 [0.90–3.31]
DE†	0.21 [0.14–0.35]	1.34 [0.65–2.48]
Non-DE	0.52 [0.34–0.77]	2.37 [1.27–3.97]
Cycle length (ms) (rate [bpm])§	161 ± 21 (379 ± 44)	1083 ± 209 (58 ± 12)

Values are given as mean ± SD, n (%), or median [interquartile range] unless otherwise indicated.

DE = delayed enhanced; LA = left atrium; MRI = magnetic resonance imaging; MRI-DE = delayed enhanced magnetic resonance imaging; pt = patient; TTE = transthoracic echocardiography.

∗ Includes subset of patients analyzed for MRI-DE vs voltage correlation.

† As determined by MRI-DE ≥2 SD above mean blood pool intensity.

‡ Eight-second acquisition, resulting in V_mAF-8_.

§ Measured from the posterior LA.

### MRI-DE

**Figure 1:**
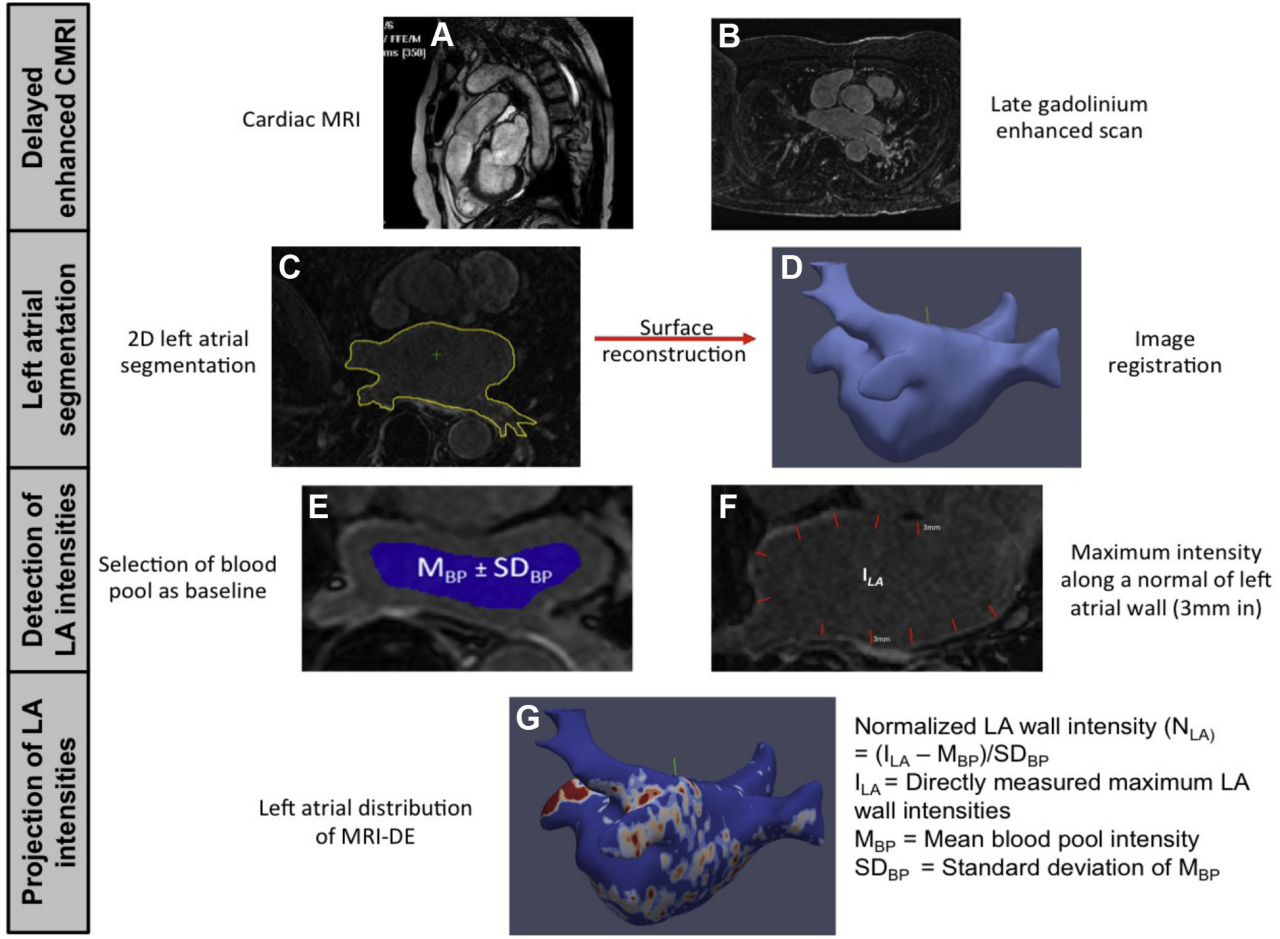
Schematic representation of the generation of left atrial (LA) delayed-enhancement magnetic resonance imaging (MRI-DE) models. 2D = 2 dimensional; MRI = magnetic resonance imaging.

All imaging was performed with a 1.5-T Philips Achieva MR system (Philips, the Netherlands) and a 5- or-32 element phased-array cardiac coil using a technique we previously reported (see Supplemental Material for details).^8^, ^9^ The workflow is summarized in Figure 1.

### Electrophysiological study

All procedures were performed after written informed consent was obtained from the patients. After transseptal puncture, electroanatomic maps (EnSite™ Velocity™, Abbott, MN) were collected, with bipolar EGM s filtered at 30–500 Hz (without 50-Hz notch filter). With operators blinded to MRI-DE results, data acquisition was performed according to study protocol before radiofrequency ablation.

### Study protocol

All patients presented in AF on the day of the procedure. Left atrial (LA) geometry and subsequent data were acquired using a double-loop catheter (AFocusII™, Abbott) with 20-ring electrodes (1-mm length, 4-mm spacing). For comparative assessment of AF vs SR voltage in the LA, baseline AF maps were collected in 14 patients via 8-second complex fractionated EGM mapping (EnSite™ Velocity™, Abbott). In a subset of 13 of 14 patients, SR was additionally maintained after external DC cardioversion, and SR voltage maps were created. Before each acquisition, the AFocusII catheter was held tangentially to the endocardial surface, enabling stable tissue contact. EGMs >5 mm from the geometry surface were automatically excluded. For subsequent quantitative analysis, all points within the pulmonary veins and LA appendage were excluded (Table 1).

### Criteria for mean AF voltage

**Figure 2:**
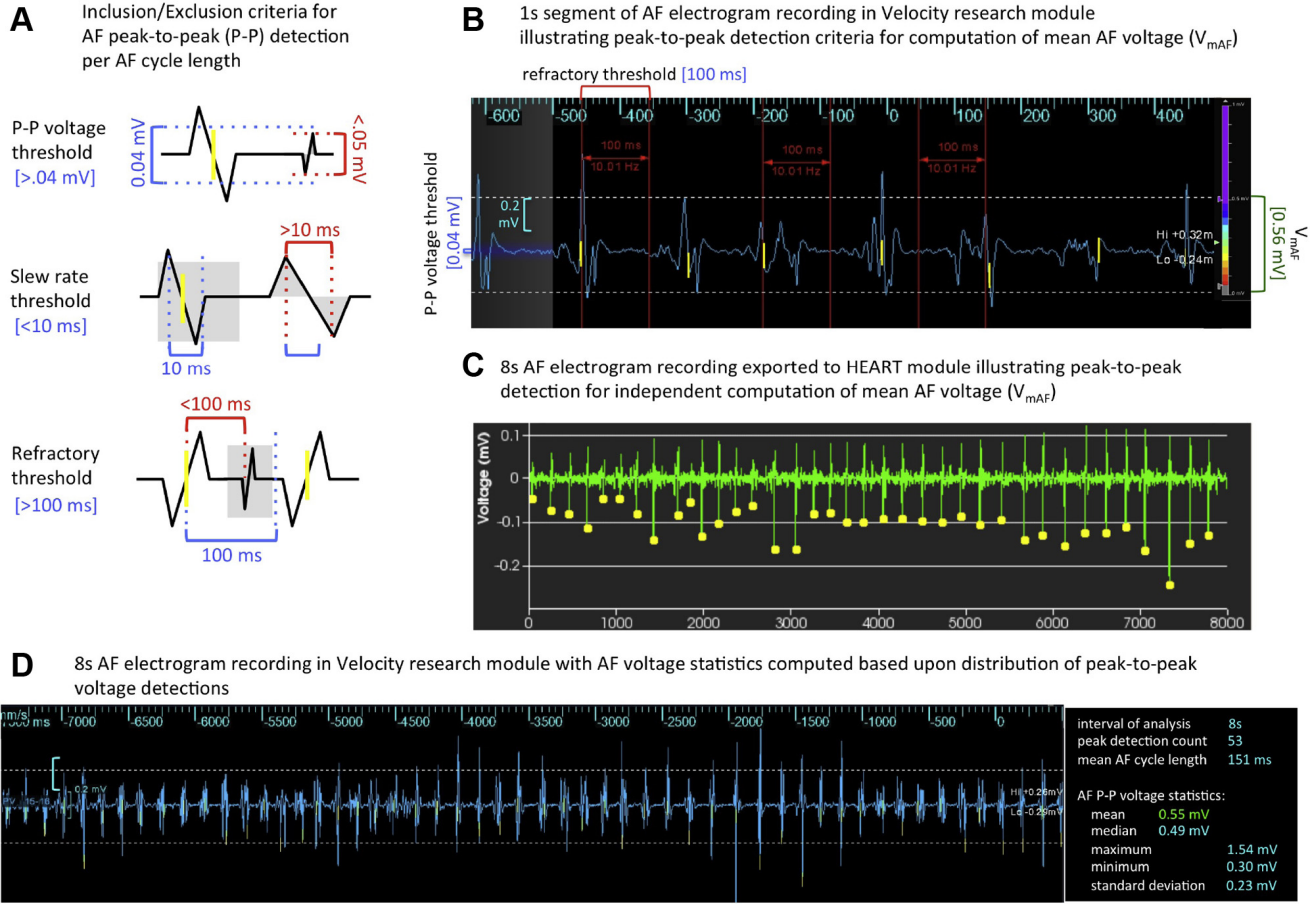
Criteria for determination of mean AF voltage (V_mAF_). **A:** Inclusion/exclusion criteria for AF peak-to-peak detection per AF cycle length. **B:** AF electrogram (1 second) illustrating implementation of P-P detection criteria in the Velocity research module. **C:** AF electrogram (8 seconds) from the HEART software module. **D:** AF electrogram (8 seconds) from the Velocity research module. AF voltage statistics are computed based on the distribution of all peak-to-peak voltage detections (**right**).

The criteria used for determination of mean AF voltage (V_mAF_) was based on detection of the maximum peak-to-peak (P-P) voltage per atrial fibrillation cycle length (AFCL), followed by statistical averaging of all AF peaks detected across the sampling interval. P-P detection criteria for any candidate deflection required a voltage threshold >0.04 mV, slew rate <10 ms, and used a 100-ms refractory window (Figures 2A and 2B). In subsequent phases of AF voltage analyses, an *index* sampling interval of 8 seconds was used as the nominal sampling interval (V_mAF-8_) (for details see Supplemental Material).

**Figure 3:**
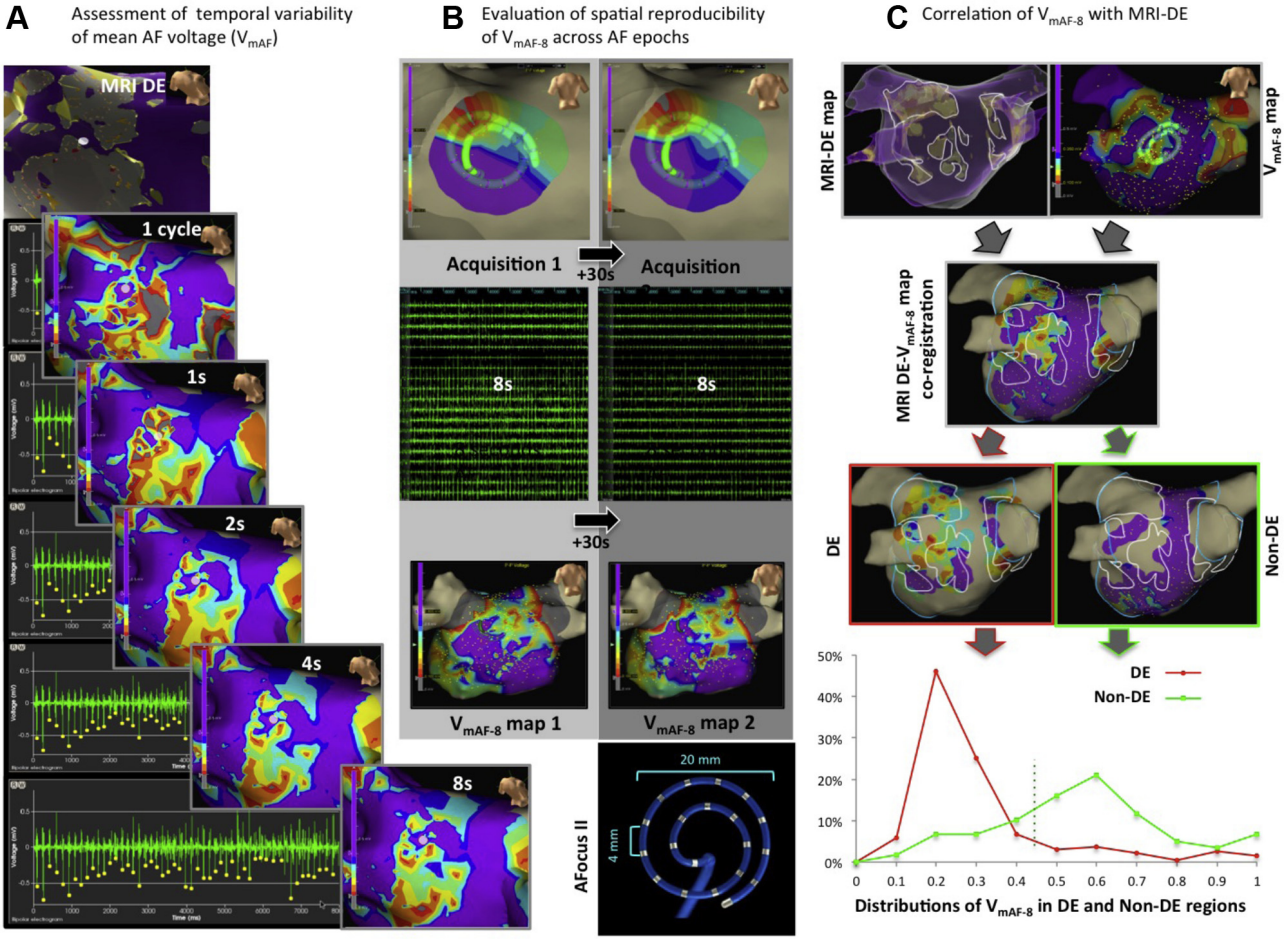
Data acquisition and analysis of mean atrial fibrillation (AF) voltage: temporal variability, spatial reproducibility, and correlation with delayed-enhancement magnetic resonance imaging (MRI-DE). **A:** Sampling adequacy AF electrograms imported into HEART. V_mAF_ per electrogram is analyzed over varying sampling intervals (1 AFCL–8 seconds). V_mAF_ maps demonstrate the spatial aspect of the temporal variability of V_mAF_ compared to the MRI-DE model (**top**). **B:** Spatial reproducibility with the *AFocusII* held in a fixed position. Two separate acquisitions are used to generate 30-second time-shifted V_mAF-8_ maps. **C:** MRI-DE correlation. The V_mAF-8_ map is coregistered with the MRI-DE map. Markers are superimposed on the V_mAF_ map delineating an MRI-DE threshold ≥2 SD M_BP_. V_mAF_ points are subregioned into DE and non-DE, allowing for comparative histogram V_mAF_ distributions. CL = cycle length.

To facilitate V_mAF_ analysis, 2 independent software modules were used: (1) HEART (High-density Electrogram Analysis Research Tool) in-house software allowed for signal analysis into the temporal variability of AF (Figure 2C); and (2) EnSite Velocity Research Module (V1.1), Abbott, MN, served as an interactive 3-dimensional platform for assessment of the spatial distribution of V_mAF_, V_SR_, and subsequent correlation with MRI-DE (Figures 2D and 3). Although the implementation details for the V_mAF_ metric varied slightly on each platform, the general methodologies used for peak detection and V_mAF_ were consistent.

### Assessing temporal variability of mean AF voltage

In 20 patients, 160 epochs of AF were recorded from the LA surface (360 ± 35 EGMs per patient; 100 ± 15 cm^2^/patient) over a 40-second duration, in order to assess the temporal variability of V_mAF_ and determine sampling adequacy. All AF segments were exported into HEART for analysis. The dominant deflection within each AFCL was detected according to the criteria described earlier, and V_mAF_ was statistically calculated over sampling intervals ranging from 0.5–8 seconds at 1113 locations (Figure 3A). The coefficient of variation of AF voltage, a measure based on the SD/mean of individual AF voltage peaks (1 peak per AFCL), was derived. Intraclass correlation coefficients (ICCs) of *sampled* V_mAF_ vs *index* V_mAF-8_ were assessed. In order to determine the minimum sampling duration sufficient to yield a statistically meaningful V_mAF_ result, the time course of V_mAF_ was generated for each EGM. Global mean voltage error (V_ME_) was computed as the difference between *sampled* V_mAF_ vs *index* V_mAF-8_. The sampling duration was determined such that V_ME_ was reduced to 0.05 mV, equivalent to the recording system noise level. The impact of sampling error on the spatial distribution of V_mAF_ was assessed (Figure 3A).

### Evaluating spatial reproducibility of mean AF voltage over time

In 15 of 20 patients, 3450 AF EGMs (244 ± 101 EGMs per patient) were acquired over a 40-second duration, enabling analysis into the spatial reproducibility of AF voltage via the EnSite Velocity research module. Time-shifted V_mAF-8_ maps were subsequently created by initiating 2 separate acquisitions per fixed catheter location separated by 30 seconds. The resulting “paired-point” V_mAF-8_ maps allowed for correlative assessment of spatial reproducibility of V_mAF-8_ across temporal epochs of AF (Figure 3B). In a subset of 2 of 15 cases, V_mAF-8_ maps were acquired sequentially, separated by a longer 20-minute waiting period. Although not a systematic study, these examples were assessed for visual evidence of spatial reproducibility of mean AF voltage.

### Correlating MRI-DE with mean AF and SR voltage

In 14 of 20 patients, spatial correlative analyses were performed for comparing distributions of low voltage during AF (V_mAF-8_) and SR (V_SR_) with regions of MRI-DE. Electroanatomic maps were coregistered with MRI-DE models using the EnSite Fusion tool in a blinded fashion,^10^ such that voltage and MRI-DE data were not projected onto the respective surfaces. LA subregions occupied by normalized intensities ≥2 SD above mean blood pool intensity (M_BP_) were assigned as delayed enhancement (DE), whereas regions with intensities <2 SD M_BP_ were categorized as normal (non-DE), consistent with previously described image intensity thresholds.^4^ Any points determined to be on the border between binarized regions were included in both subgroups (Figure 3C).

Distributions for both V_mAF-8_ (4279 EGM sites) and V_SR_ (3694 EGM sites) were sampled from both DE and non-DE regions of the LA posterior wall (Table 1). Comparative voltage distribution curves were generated for both V_mAF-8_ and V_SR_ in DE vs non-DE regions (Figure 3C, bottom). Receiver operating characteristic (ROC) curves were derived to assess sensitivity/specificity of V_mAF-8_ and V_SR_ for detection of MRI-DE, allowing for determination of optimal voltage cutoffs.

### Statistical analysis

All normal variables are expressed as mean ± SD. The Shapiro-Wilk test was used to determine the normality of the distributions of voltages. Nonparametric distributions of V_mAF-8_ and V_SR_ are given as median (interquartile range [IQR]). Paired and unpaired *t* tests were used when appropriate, with Bonferroni correction for multiple tests. Correlation of continuous variables was examined with Pearson and Spearman correlation coefficients for parametric and nonparametric data respectively. ROC curves were compared using the DeLong test. P <.05 was considered significant.

## Results

Patient characteristics are listed in Table 1.

### Sampling adequacy of mean AF voltage

The global coefficient of variation of P-P AF voltage was 45% for 2 sampled AFCLs. Sampling 10, 20, and 40 AFCLs reduced coefficient of variation to 18%, 12%, and 9%, respectively.

**Figure 4:**
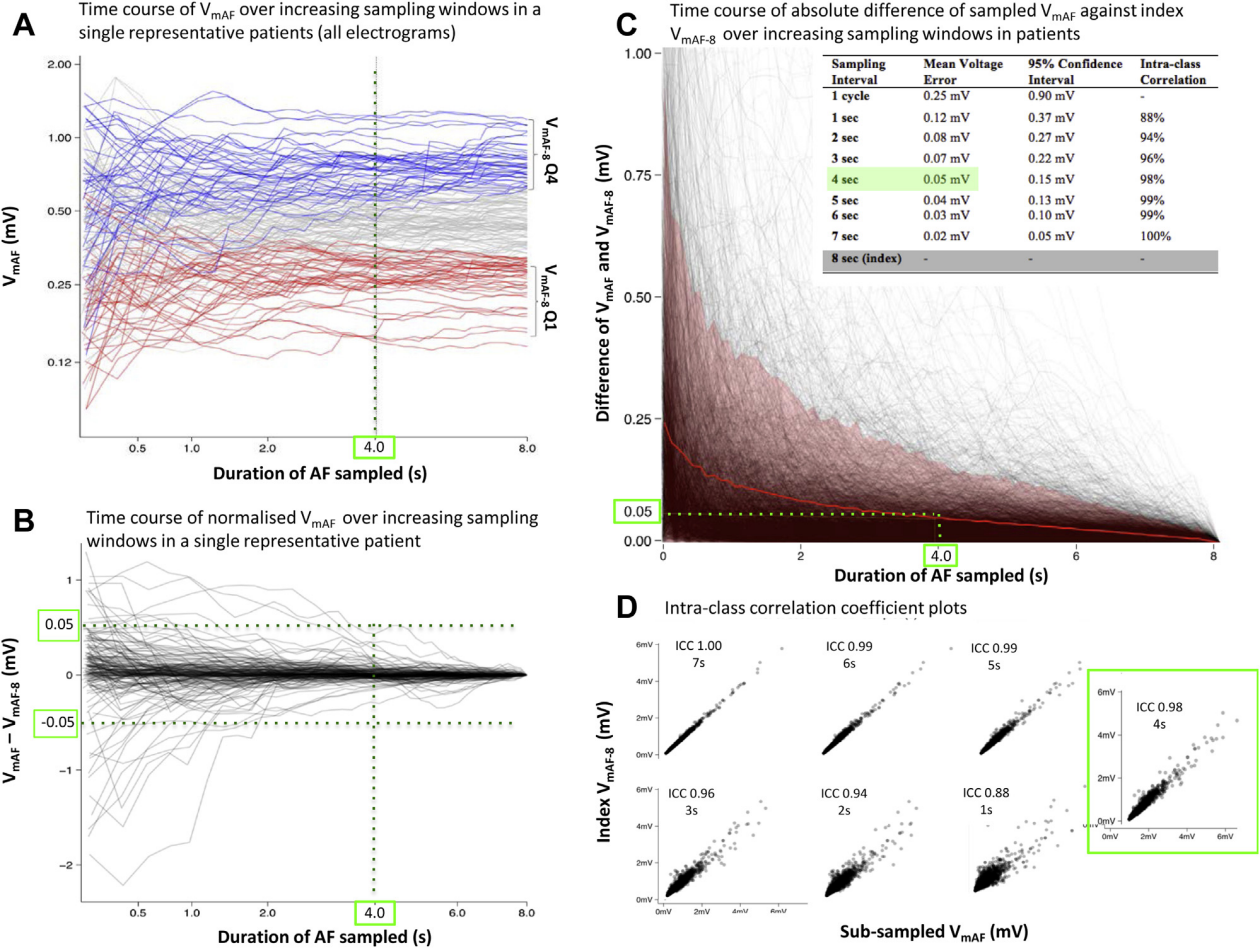
Assessment of the temporal stability of atrial fibrillation (AF) voltage. **A:** Time course of V_mAF_ from a single patient. Traces in the top and bottom quartiles of V_mAF_ are colored *blue* and *red*, respectively. By 4 seconds, crossover of V_mAF_ between the top and bottom quartiles for all traces has occurred, as mean V_AF_ has stabilized. **B:** Normalized time course of V_mAF_ (from traces in panel A). By 4 seconds, none of the traces deviate from the index 8-second mean by more than *±*0.05 mV. **C:** Global time course of the absolute difference between sampled and index 8-second mean AF voltage (*black lines*). Traces within the *pink* confines represent electrograms in the upper 95% confidence interval. *Red line* represents the mean voltage error (V_ME_) of V_mAF_. **D:** Intraclass correlation (ICC) plots of 8-second index mean V_AF_ (y-axis) vs subsampled V_mAF_ values for sampling intervals ranging from 1–7 seconds (x-axis).

In representative data from a single patient, individual V_mAF_ traces are plotted as a function of time, indicating that beyond 4 seconds, V_mAF_ has stabilized (Figures 4A and 4B). Global results (3450 EGMs) quantifying the trend of V_mAF_ stabilization are presented graphically, showing that as the sampling window reaches 4 seconds, V_ME_ approaches 0.05 mV (Figure 4C). Sampling beyond 4 seconds yields diminishing returns, with V_ME_ lowered ∼0.01 mV for each additional 1 second of sampling (Figure 4C, inset).

Global ICCs of *sampled* V_mAF_ vs *index* V_mAF-8_ show improving correlation with increased sampling. Increasing the sampling window in 1-second increments (1–7 seconds) yields respective ICC values of 0.88, 0.94, 0.96, 0.98, 0.99, 0.99, and 1.00 (Figure 4D).

The spatial impact of sampling duration on V_mAF_ is shown in Figure 3A. In this representative case, the spatial distribution of V_mAF_ was rendered at sampling intervals ranging from 1 AFCL–8 seconds. Beyond a sampling interval of 4 seconds, the V_mAF_ maps were visually similar, matching the MRI-DE distribution.

### Spatial reproducibility of mean AF voltage across epochs

**Figure 5:**
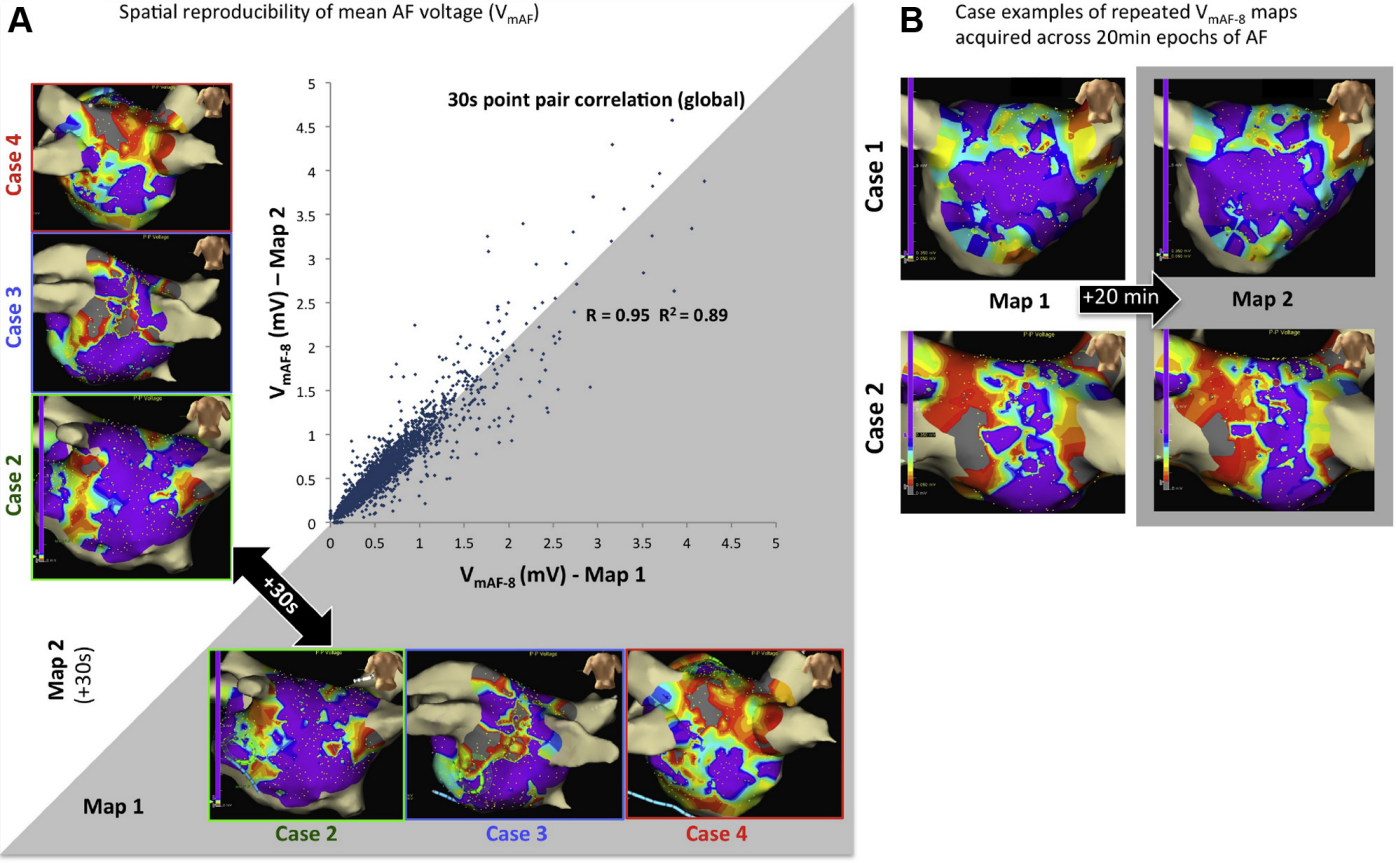
Spatial reproducibility of mean atrial fibrillation (AF) voltage. **A:** Global correlation (15 patients; 3450 locations) of V_mAF_ point-pairs acquired 30 seconds apart. Cases 2, 3, and 4 demonstrate congruency of the individual maps. **B:** Two case examples of 8-second V_mAF_ maps (5-mm interpolation) acquired sequentially, separated by a 20-minute waiting period.

Time-shifted (30 seconds) V_mAF-8_ maps revealed visually similar spatial distributions. Global analyses of all point-pairs (3450 EGM locations; n = 15) resulted in a high degree of correlation between the 2 distinct temporal epochs of AF (Pearson correlation coefficient r = 0.95; r^2^ = 0.89; *P* <.001) (Figure 5A). Additionally, 2 sequentially acquired V_mAF-8_ maps, separated by a 20-minute waiting period, illustrate visual similarity in V_mAF_ distribution (Figure 5B).

### MRI-DE correlation with mean AF vs SR voltage

Global median voltage was 0.35 [0.19–0.61] mV during AF vs 1.81 [0.90–3.31] mV during SR, resulting in V_mAF_/V_SR_ ratio of 1:5.18 (19.3%) (Table 1).

**Figure 6:**
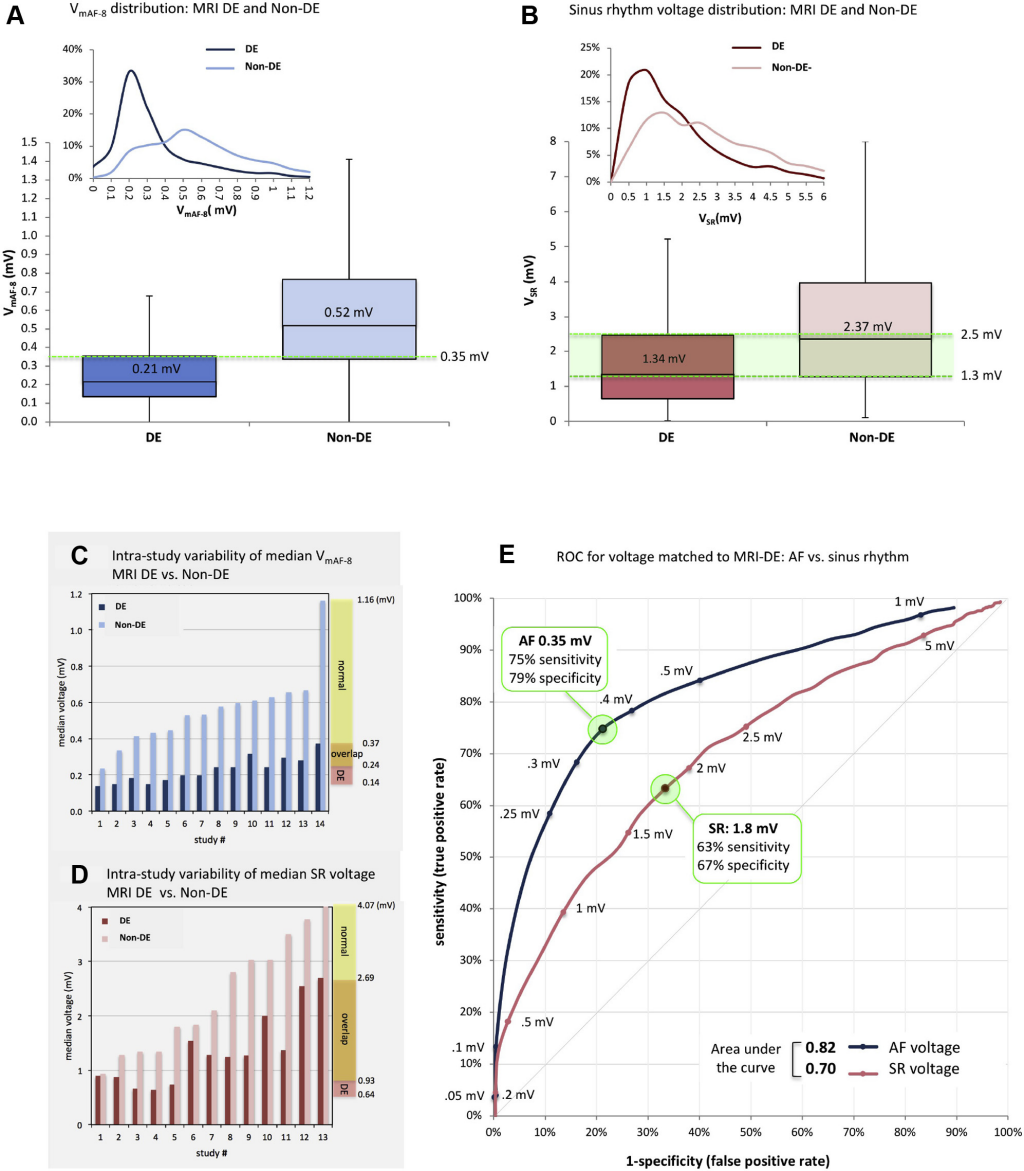
Distributions of 8-second mean atrial fibrillation(AF) and sinus rhythm (SR) voltage in delayed enhanced (DE) vs non–delayed enhanced (non-DE) subregions. **A:** Global distribution of V_mAF-8_ in DE vs non-DE subregions. **B:** Global distribution of V_SR_ in DE vs non-DE subregions. **C:** Intrastudy variability of median V_mAF-8_ in DE vs non-DE subregions across patients. **D:** Intrastudy variability of median V_SR_ in DE vs non-DE subregions across patients. **E:** Receiver operating characteristic (ROC) curves for respective AF and SR voltage match to delayed-enhancement magnetic resonance imaging (MRI-DE).

During AF, voltage distributions in DE vs non-DE regions were 0.21 [0.14–0.35] mV vs 0.52 [0.34–0.77] mV, respectively (*P* <.0001), showing minimal overlap in the intraquartile range (0.34–0.35 mV) (Figure 6A). During SR, voltage distributions in DE vs non-DE regions were 1.34 [0.65–2.48] mV vs 2.37 [1.27–3.97] mV, respectively (*P* <.0001), showing a high degree of intraquartile overlap (1.3–2.5 mV) (Figure 6B).

Intrapatient variability of median AF voltage (n = 14) ranged from 0.14–0.37 mV in DE vs 0.24–1.16 mV in non-DE. Median AF voltage <0.24 mV was specific to the DE subgroup, whereas >0.37 mV was specific to the non-DE subgroup (Figure 6C). During SR (n = 13), voltage ranges were 0.64–2.69 mV in DE vs 0.93–4.07 mV in non-DE. Median SR voltage <0.93 mV was specific to DE, whereas >2.69 mV was specific to non-DE (Figure 6D).

### Threshold discrimination of AF vs SR to match MRI-DE

ROC results for threshold discrimination of AF and SR voltage to match MRI-DE are shown in Figure 6E.

V_mAF-8_ detected for the presence of MRI-DE with sensitivity of 75% and specificity of 79% at voltage cutoff of 0.35 mV and area under the curve (AUC) of 0.82. A cutoff range of 0.3–0.4 mV preserved balanced sensitivity/specificity tradeoffs of 68%/84% at 0.3 mV and 78%/73% at 0.4 mV. V_SR_ detected for the presence of MRI-DE with sensitivity of 63% and specificity of 67% at voltage cutoff of 1.8 mV and AUC of 0.70. Legacy V_SR_ thresholds 0.5–1 mV resulted in high specificity (97% at 0.5 mV, 86% at 1 mV) but low sensitivity (18% at 0.5 mV, 39% at 1 mV) for detection of MRI-DE. AUCs were highly statistically significant (*P* <.0001).

**Figure 7:**
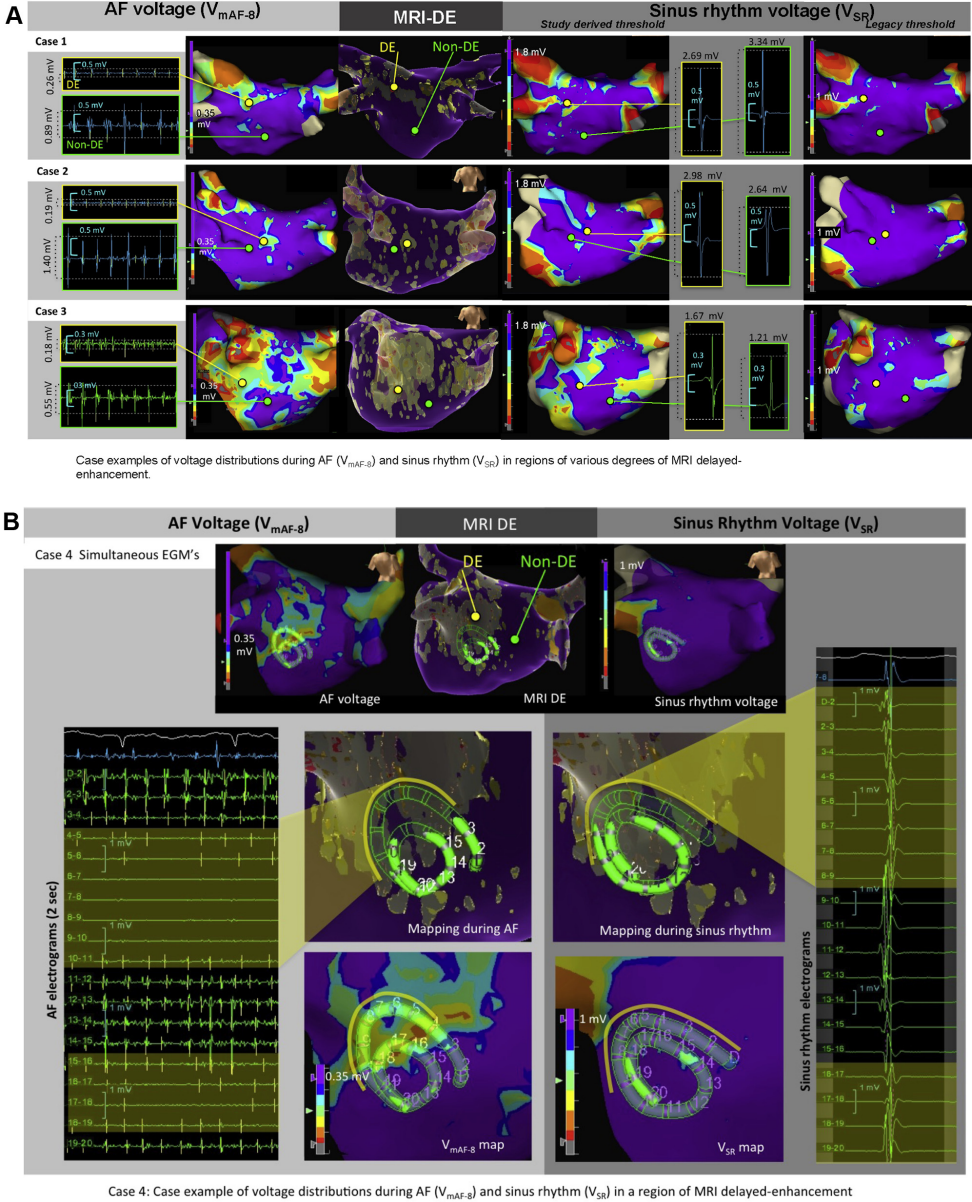
Case examples of atrial fibrillation (AF) and sinus rhythm (SR) voltage vs delayed-enhancement magnetic resonance imaging (MRI-DE). **A:** Case examples of AF (V_mAF-8_) and SR voltage with varying degrees of MRI-DE–detected fibrosis. In all cases, the AF voltage map (**left**) (cutoff range *0.05–0.35 mV*) provides a visual spatial match with MRI-DE (>2 SD M_BP_). SR voltage maps set to the legacy cutoff range (0.1–1.0 mV; **right**) lack the sensitivity to detect MRI-DE. As the V_SR_ threshold is increased to the study-derived threshold (1.8 mV; **middle**), sensitivity for MRI-DE detection is improved, whereas specificity is reduced. Representative electrograms are displayed from both DE/non-DE regions of each map. **B:** Case example with simultaneous electrograms sampled across the DE/non-DE border during both AF (**left**) and SR (**right**). During AF, the transition from non-DE to DE can be seen in both the differential amplitude of electrograms and in the V_mAF-8_ map (cutoff range *0.05–0.35 mV*). In contrast, electrograms during SR reveal no obvious differential. The V_SR_ map (cutoff range 0.1–1.0 mV) has no low-voltage zones.

The clinical impact of these results is shown in 4 case examples in which AF voltage (0.35-mV cutoff) can be visually appreciated as a better match to regions of MRI-DE than SR voltage (1.0-mV cutoff) (Figure 7).

## Discussion

### Main findings

Under conditions of adequate sampling (≥4 seconds), mean AF voltage is a stable, reproducible metric, yielding high sensitivity and specificity to MRI-DE as assessed from the posterior LA of our persistent AF cohort. SR voltage correlates poorly to MRI-DE, with legacy thresholds (0.5–1 mV) yielding good specificity but unacceptably low sensitivity to the *de novo* substrate as detected by MRI-DE.

### Implications for legacy SR voltage thresholds as a marker for fibrosis

Previous studies have suggested that SR voltages <0.5–1.0 mV represent varying degrees of “diseased” regions^5^, ^6^ and correlate with MRI-DE.^2^, ^8^, ^9^ We showed that V_SR_ threshold <1.0 mV failed to demonstrate meaningful sensitivity to regions enhanced by MRI-DE. By applying a 95% normal threshold criteria, Kapa et al^11^ prescribed a conservative 0.2- to 0.45-mV V_SR_ threshold range for the detection of atrial scar. Applying Kapa’s thresholds to our own ROC results, V_SR_ was found to be highly specific (>97%) but insensitive (5%–15%) to the extent of *de novo* fibrosis as detected by MRI-DE. Any attempt to increase sensitivity by raising thresholds (eg, 2.0 mV: 67%; 2.5 mV: 75%) resulted in an unacceptable decrease in specificity (2.0 mV: 62%; 2.5 mV: 51%), increasing the likelihood of false-positive detection of normal tissue. We theorize that the difference between the Kapa V_SR_ cutoffs and our own may lie in the fact that their cohort included patients with ablation-induced scar and was not exclusive to the *de novo* substrate.

### AF voltage for identification of the arrhythmogenic substrate

Bipolar voltage has long been accepted for detection of scar in the context of ventricular tachycardia.^12^ More recently, low-voltage zones during SR^5^, ^6^ and AF^13^ have been targeted in patients with persistent AF, resulting in improved clinical outcomes.

During AF, slow conduction in diseased myocardium may be necessary for sustaining rotational activity, whether localized or macroreentrant. Regions of rotational activity have been shown to colocalize with low-voltage regions (<0.1 mV) during AF in some studies^14^ but not in others.^15^, ^16^

MRI-defined burden of atrial fibrosis correlates with clinical indices of structural remodeling and outcomes of catheter ablation.^17^ Sites exhibiting high rotational activity have shown clustering around borders of fibrotic areas in noninvasive mapping.^18^ The DECAAF (Delayed-Enhancement MRI Determinant of Successful Radiofrequency Catheter Ablation of Atrial Fibrillation) Investigators^17^ defined “residual fibrosis” as areas of overlap between ablation-induced scar with *de novo* fibrosis and retrospectively showed that patients who had less “residual fibrosis” had improved outcomes after ablation.^19^ These observations formed the basis of the DECAAF-2 study, in which regions of MRI-defined fibrosis are targeted during catheter ablation for AF.

### Pathophysiological basis of voltage during AF

The relationship between AF and the underlying setting of fibrosis is unclear. Pathophysiologically, fibrosis can present in a spectrum of textures, resulting in distinct arrhythmogenic profiles. *Compact* fibrosis comprises dense regions of collagen that are readily resolved by MRI, electrophysiologically resulting in low voltage associated with fixed conduction block. *Noncompact* fibrosis (interstitial/reparative) encompasses more diffuse textures at or below the margins of current MRI resolution and is associated with functional arrhythmogenic vulnerability. Interstitial fibrosis involves collagenous separation of muscle bundles, resulting in slow conduction and functional reentry, whereas reparative fibrosis involves diffuse cardiomyocyte replacement, rendering tissue susceptible to anisotropy and refractory dispersion.^1^, ^20^, ^21^

During SR, the underlying AF substrate is in an electrophysiologically passive state, featuring low rates and coordinated activation wavefronts. Under these conditions, low voltage is known to result as the propagating wavefront encounters conduction barriers associated with *compact* fibrosis. In this passive state, *noncompact* fibrotic regions susceptible to arrhythmogenic activity may lie dormant.

The effects of arrhythmia function on voltage are less well understood. In a series comparing voltage amplitudes during SR vs atrial flutter, Bradfield et al^22^ observed the discordance of voltage associated with functional effects of the underlying rhythm. In their series, rhythm-based voltage differences were attributed to rate/wavefront direction, resulting in variability of functional block. In a controlled pacing study, Iso et al^23^ further elaborated on the functional influences on LA voltage, finding site-specific EGM amplitudes to be both rate and direction dependent.

During AF, activation rates are more rapid than those associated with any organized rhythm. Ndrepepa et al^24^ reported on the association between regional AFCL and AF voltage, observing the greatest AF voltage reductions occur in regions of “faster and more disorganized activity.” Accordingly, we theorize that the higher intrinsic rates present during AF may be associated with underlying *noncompact* fibrotic regions vulnerable to slow conduction and functional reentry, resulting in the manifestation of low voltage.

Furthermore, during AF, regional wavefront multiplicities may arise in the wake of conduction disturbances resulting from tissue anisotropies and refractory dispersion in areas of *noncompact* fibrosis. Importantly, such wavefront scatter is less likely to present during SR, which could further explain SR’s poor voltage sensitivity to *noncompact* fibrosis. In a supporting finding, Masuda et al^25^ demonstrated that although colocalized AF and SR EGM voltage amplitudes were well correlated in locations with *normal* EGM morphologies, the correlation disappeared at locations where *normal* EGMs during SR became *fractionated* during AF.

In this series, we reported that AF voltage yielded significantly higher sensitivity and specificity to MRI-detected fibrosis than SR voltage. We theorize that although either rhythm condition may elicit a low-voltage response to *compact* fibrosis, the difference may lie in the voltage response to the *noncompact* spectrum of fibrosis. We propose that arrhythmogenic vulnerabilities (eg, conduction slowing, functional reentry, anisotropy, refractory dispersion) associated with the *noncompact* spectrum of fibrosis, although *dormant* during SR, become *manifest* during the functional circumstances encompassing AF. As susceptible regions are "activated," low voltage is an expression of the underlying electro-architectural substrate that can only be elucidated under conditions of electrophysiological stress. AF, being the clinical rhythm of interest, conveniently provides the ideal setting to explore this paradigm.

### Study limitations

MRI-DE for detection of atrial fibrosis has faced criticism for its lack of resolution, susceptibility to artifact, and lack of standardization.^4^ It is important to note that current MRI resolution may not allow for the detection of more diffuse fibrotic change and also precludes the study of nontransmural (endo–epicardial) fibrosis. Although our imaging results have been validated for ablation-induced scar^8^, ^9^ and reflect *relative* spatial distributions of LA MRI-DE, we acknowledge that reproducing *absolute* image intensity results across centers depends on increased standardization of MRI-DE postprocessing methodologies.

Absolute voltage thresholds are dependent on the bipole configuration of the mapping catheter used,^26^ in addition to other factors such as bipole orientation/wavefront direction and filtering.^7^ We elected to use the AFocusII™ catheter (1-mm electrodes, 4-mm spacing), which allowed for high sampling density and stable tissue contact. We believe the significance of our results lies less in the prescription of absolute voltage thresholds and more in the *relative* nature of the relationships established between AF/SR voltage and MRI-detected fibrosis.

We acknowledge that some of the discrepancy between SR voltage and MRI-DE may potentially result from the delay in electrical recovery after acute cardioversion from long-standing persistent AF.^27^

Several groups have reported on the intrinsic variability of atrial voltage amplitudes with anatomic location.^11^ In order to obtain highest fidelity EGMs, our electroanatomic data were limited to the posterior LA, which was (1) anatomically consistent, (2) conducive to placement of the mapping catheter tangent to the endocardial surface, and (3) contained a predilection of fibrosis. This choice allowed us to best meet the objective of assessing the relationship between voltage and fibrosis. However, we accept the limitation that the LA posterior wall does not comprise the global persistent AF substrate and that other regions of the left and right atria should be considered in future studies.

## Conclusion

Mean AF voltage, when sampled for ≥4 seconds, is a statistically robust and reproducible metric as assessed in the *de novo* persistent AF substrate. Within the posterior LA, AF voltage, to a greater extent than SR voltage, is both sensitive and specific for detection of MRI-DE. Although legacy SR voltage thresholds (0.5–1 mV) exhibit poor sensitivity to MRI-DE, we theorize that AF voltage is sensitive to the substrate that can only be elucidated under conditions of functional electrophysiological stress. Assessment of mean AF voltage can potentially be implemented widely, which would enable broader investigation into its utility as a surrogate for fibrosis, potentially obviating the need for MRI-DE, in characterizing the structural arrhythmic substrate.

## References

[1] Iwasaki Y.-K., Nishida K., Kato T., Nattel S. (2011). Atrial fibrillation pathophysiology: implications for management. Circulation.

[2] Oakes R.S., Badger T.J., Kholmovski E.G. (2009). Detection and quantification of left atrial structural remodeling with delayed-enhancement magnetic resonance imaging in patients with atrial fibrillation. Circulation.

[3] Akoum N., Wilber D., Hindricks G. (2015). MRI Assessment of ablation-induced scarring in atrial fibrillation: analysis from the DECAAF Study. J Cardiovasc Electrophysiol.

[4] Benito E.M., Carlosena-Remirez A., Guasch E. (2017). Left atrial fibrosis quantification by late gadolinium-enhanced magnetic resonance: a new method to standardize the thresholds for reproducibility. Europace.

[5] Kottkamp H., Berg J., Bender R., Rieger A., Schreiber D. (2015). Box isolation of fibrotic areas (BIFA): a patient-tailored substrate modification approach for ablation of atrial fibrillation. J Cardiovasc Electrophysiol.

[6] Yang G., Yang B., Wei Y., Zhang F., Ju W. (2016). Catheter ablation of nonparoxysmal atrial fibrillation using electrophysiologically guided substrate modification during SR after pulmonary vein isolation. Circ Arrhythm Electrophysiol.

[7] Josephson M.E., Anter E. (2015). Substrate mapping for ventricular tachycardia. JACC Clin Electrophysiol.

[8] Malcolme-Lawes L.C., Juli C., Karim R. (2013). Automated analysis of atrial late gadolinium enhancement imaging that correlates with endocardial voltage and clinical outcomes: a 2-center study. Heart Rhythm.

[9] Hunter R.J., Jones D.A., Boubertakh R. (2013). Diagnostic accuracy of cardiac magnetic resonance imaging in the detection and characterization of left atrial catheter ablation lesions: a multicenter experience. J Cardiovasc Electrophysiol.

[10] Brooks A.G., Wilson L., Kuklik P. (2008). Image integration using NavX Fusion: initial experience and validation. Heart Rhythm.

[11] Kapa S., Desjardins B., Callans D.J., Marchlinski F.E., Dixit S. (2014). Contact electroanatomic mapping derived voltage criteria for characterizing left atrial scar in patients undergoing ablation for atrial fibrillation. J Cardiovasc Electrophysiol.

[12] Marchlinski F.E., Callans D.J., Gottlieb C.D., Zado E. (2000). Linear ablation lesions for control of unmappable ventricular tachycardia in patients with ischemic and nonischemic cardiomyopathy. Circulation.

[13] Jadidi A.S., Lehrmann H., Keyl C. (2016). Ablation of persistent atrial fibrillation targeting low-voltage areas with selective activation characteristics. Circ Arrhythm Electrophysiol.

[14] Ghoraani B., Dalvi R., Gizurarson S. (2013). Localized rotational activation in the left atrium during human atrial fibrillation: relationship to complex fractionated atrial electrograms and low-voltage zones. Heart Rhythm.

[15] Narayan S.M., Shivkumar K., Krummen D.E., Miller J.M., Rappel W.J. (2013). Panoramic electrophysiological mapping but not electrogram morphology identifies stable sources for human atrial fibrillation. Circ Arrhythm Electrophysiol.

[16] Schade A., Nentwich K., Costello-Boerrigter L.C. (2016). Spatial relationship of focal impulses, rotors and low voltage zones in patients with persistent atrial fibrillation. J Cardiovasc Electrophysiol.

[17] Marrouche N.F., Wilber D., Hindricks G. (2014). Association of atrial tissue fibrosis identified by delayed enhancement MRI and atrial fibrillation catheter ablation: the DECAAF study. JAMA.

[18] Cochet H., Dubois R., Yamashita S. (2018). Relationship Between fibrosis detected on late gadolinium-enhanced cardiac magnetic resonance and re-entrant activity assessed with electrocardiographic imaging in human persistent atrial fibrillation. JACC Clin Electrophysiol.

[19] Akoum N., Daccarett M., McGann C. (2011). Atrial fibrosis helps select the appropriate patient and strategy in catheter ablation of atrial fibrillation: a DE-MRI guided approach. J Cardiovasc Electrophysiol.

[20] Nguyen T.P., Qu Z., Weiss J.N. (2014). Cardiac fibrosis and arrhythmogenesis: the road to repair is paved with perils. J Mol Cell Cardiol.

[21] McDowell K.S., Arevalo H.J., Maleckar M.M., Trayanova N.A. (2011). Susceptibility to arrhythmia in the infarcted heart depends on myofibroblast density. Biophys J.

[22] Bradfield J.S., Huang W., Tung R. (2013). Tissue voltage discordance during tachycardia versus sinus rhythm: implications for catheter ablation. Heart Rhythm.

[23] Iso K., Watanabe I., Kogawa R. (2017). Wavefront direction and cycle length affect left atrial electrogram amplitude. J Arrhythm.

[24] Ndrepepa G., Schneider M.A.E., Karch M.R. (2003). Impact of atrial fibrillation on the voltage of bipolar signals acquired from the left and right atria. Pacing Clin Electrophysiol.

[25] Masuda M., Fujita M., Iida O. (2017). Comparison of left atrial voltage between sinus rhythm and atrial fibrillation in association with electrogram waveform. Pacing Clin Electrophysiol.

[26] Tung R., Kim S., Yagishita D. (2016). Scar voltage threshold determination using ex vivo magnetic resonance imaging integration in a porcine infarct model: Influence of interelectrode distances and three-dimensional spatial effects of scar. Heart Rhythm.

[27] Nishino M., Hoshida S., Tanouchi J. (2000). Time to recover from atrial hormonal, mechanical, and electrical dysfunction after successful electrical cardioversion of persistent atrial fibrillation. Am J Cardiol.

