# Supplementary material for: Voltage during atrial fibrillation is superior to voltage during sinus rhythm in localizing areas of delayed enhancement on magnetic resonance imaging: An assessment of the posterior left atrium in patients with persistent atrial fibrillation
Source: Heart Rhythm. 2019 Sep;16(9):1357–67. doi: 10.1016/j.hrthm.2019.05.032 (PMC6722483; doi:10.1016/j.hrthm.2019.05.032)
Supplement: Supplemental Material [file mmc1.docx]

**Supplemental material**

**Methods**

***Delayed-enhancement magnetic resonance imaging***

All imaging was performed using a 1.5T Philips Achieva MR system and a 5-or-32 element phased-array cardiac coil providing voxel resolution of *1.5x1.5x4mm*, reconstructed to *1.25x1.25x2mm*. The exact MRI-DE sequence has previously been described in our previous work on post-ablation scar(1). Patients were in sinus rhythm or rate-controlled AF to ensure optimal image quality, and patients with poor quality scans as determined by an experienced cardiac radiologist were excluded from the study. Left atrial (LA) segmentation was manually performed on ITK-SNAP(2), which involved the delineation of the atrial epicardial border on the DICOM images derived from the MRI-DE sequences. Semi-automated software written in C++ assisted in performing rigid registration between the segmented LA and MRI-DE surfaces. The LA blood pool was used as a non-enhancing region against which the LA wall enhancement could be compared and normalized. The blood pool was identified automatically by shrinking the LA segmentation using mathematical morphology, and mean (M_BP_) and standard deviation (SD_BP_) intensity of the blood pool were calculated. Maximum LA wall intensity (I_LA_) was determined along the normal to the wall at each location, *3mm* inside the LA surface to allow for wall thickness and to exclude detection of enhanced extra-cardiac structures such as the esophagus and descending aorta. LA wall intensities were expressed as multiples of SDBP above the blood pool mean to provide a normalized LA wall intensity (N_LA_), such that *N_LA_=(I_LA_–M_BP_)/SD_BP_* (Figure 1).


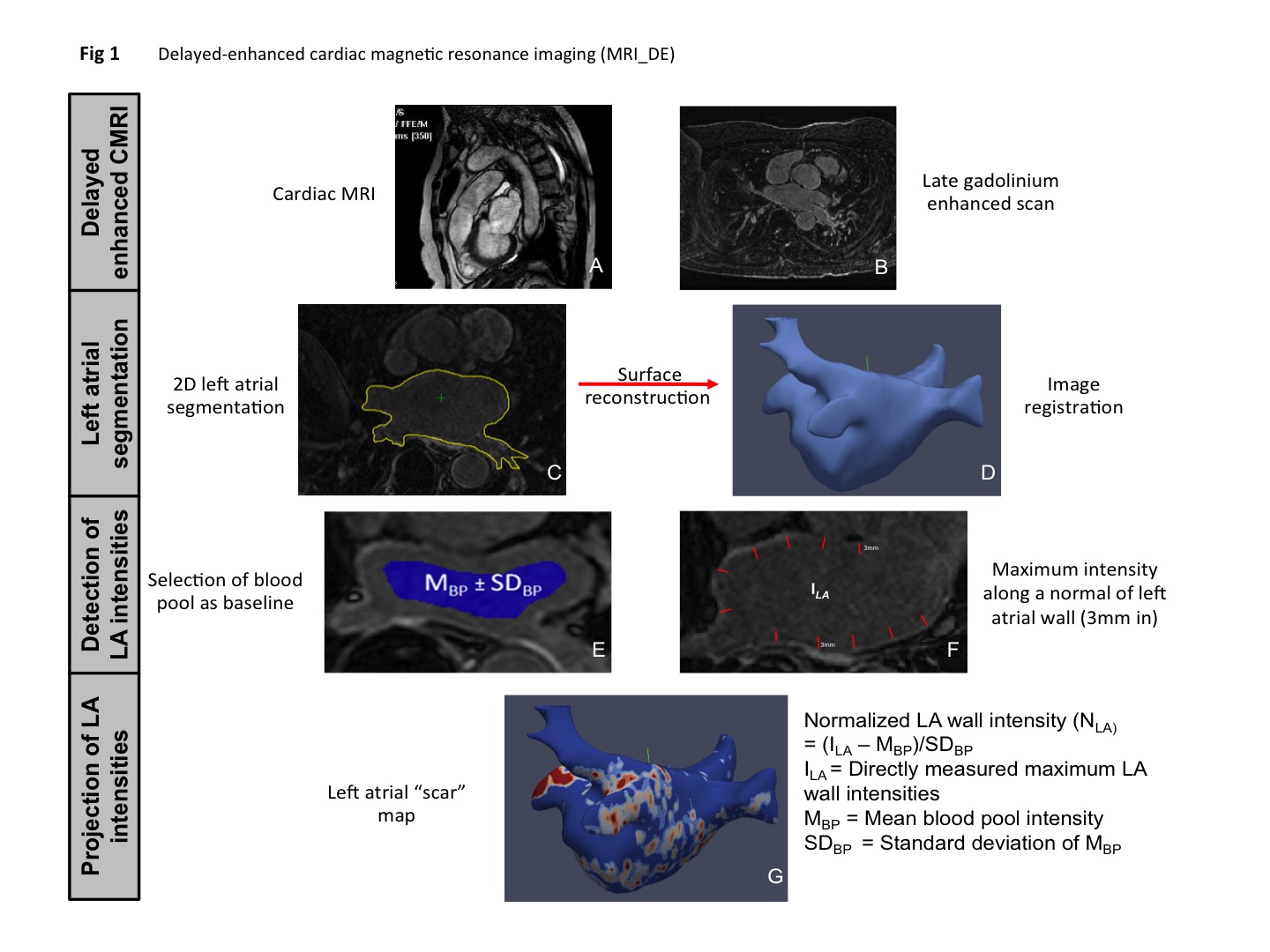


**Figure 1.** **Schematic representation of the generation of the left atrial “fibrosis” maps from MRI-DE**

***Criteria for mean AF voltage: electrogram annotation***

As assessed in the time domain of the AF electrogram, all positive and negative (+/-) candidate peaks were initially identified within a moving window. Paired +/- or -/+ peaks were classified as detection candidates if they 1) occurred within *25ms* of each other, 2) met a voltage threshold of at least *0.05mV* chosen according to the baseline noise level (Figure 2A), and 3) achieved a sufficient slew rate *(10ms)* chosen to minimize far field detection (Figure 2B). A refractory window *(100ms)* was applied at the point of the first peak-pair detection, chosen to match the minimum expected intrinsic AFCL, thus minimizing double counting of detections per AFCL (Figure 2C-E). In the case that a larger deflection was subsequently encountered within the blanking window, it was selected in place of the initial deflection and used as the starting point for the next refractory window (Figure 2F). With each detection per AFCL assigned an individual P-P value, a statistical mean voltage V_AF_ was then computed based upon all peak detections across the sampling window (Figure 2H). In all phases of subsequent AF voltage analyses performed in this study, an *index* sampling duration of *8s* was treated as the nominal "gold standard" for AF sampling interval (V_mAF-8_).


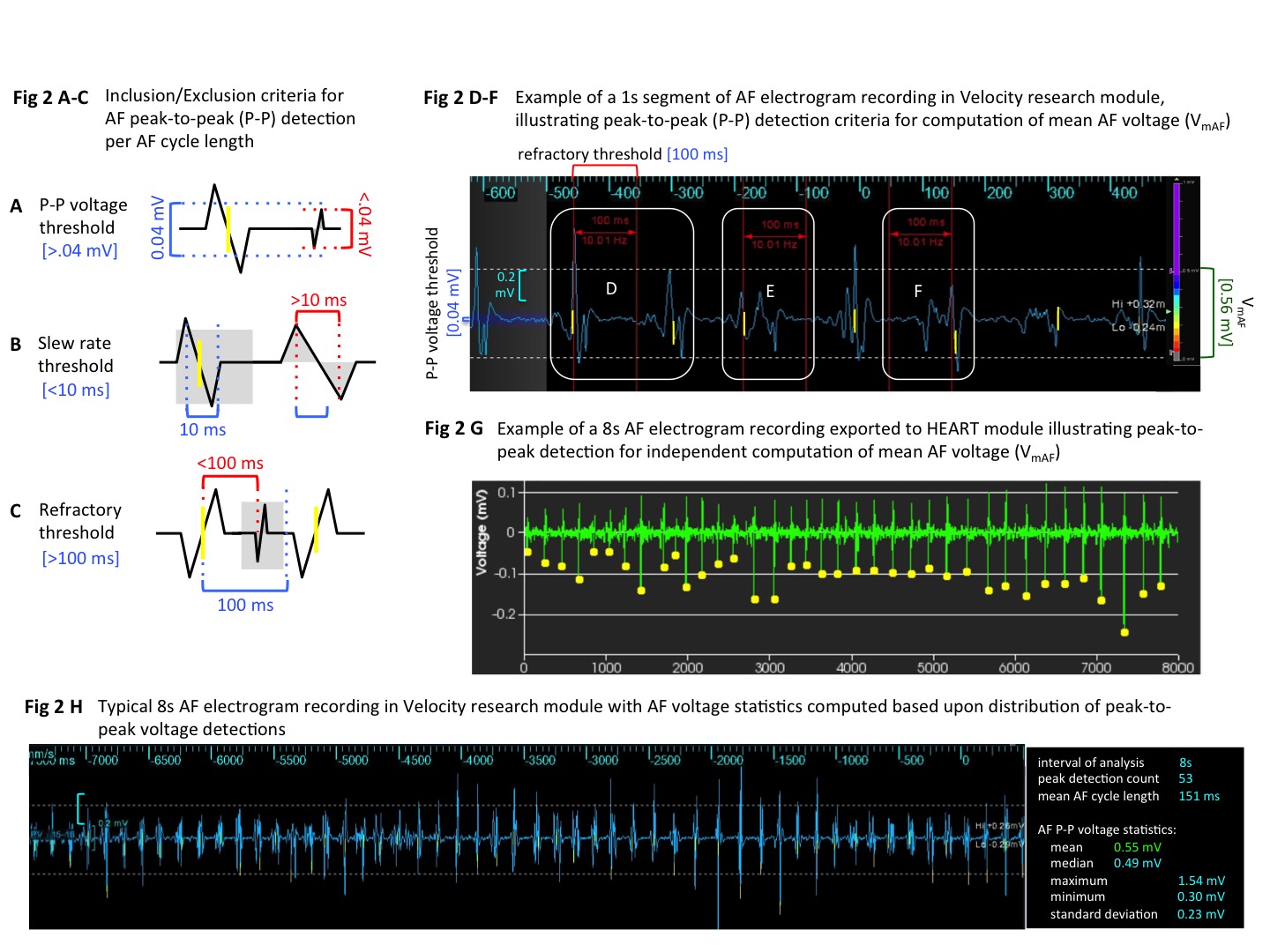


**Figure 2. Criteria for the determination of mean AF voltage (V_mAF_)**

**(Fig 2A-C)** Schematic of inclusion/exclusion criteria for detection of individual voltage peaks within the AF electrogram, according to (A) voltage threshold, (B) slew rate threshold, and (C) refractory threshold.

(**Fig 2D-F)** AF electrogram (1sec) from the *Velocity research* module. In (D) both voltage peaks are detected (yellow marks) as each meets all inclusion criteria outlined in (A-C). In (E), the second peak is not detected, as it falls within the 100ms refractory window. In (F), while both deflections fall within the same refractory window, the second deflection is detected due to larger voltage amplitude.

**(Fig 2G)** AF electrogram (8sec) from the *HEART* *software* module. Voltage peaks are detected (yellow marks) according to the criteria detailed in (A-C).

**(Fig 2H)** AF electrogram (8sec) from the *Velocity research* module with AF voltage statistics computed (right) based upon the distribution of all peak-to-peak voltage detections.

**Results**

***Evaluating spatial reproducibility of mean AF voltage across epochs***

All patients underwent mean AF voltage mapping during separate epochs of AF separated by 30s. Electrogram data was collected over an approximately 40s duration, i.e. there was 30s intervening between the start of the acquisition of the first map, to the beginning of the second sampled map and point pair analysis was performed (Figure 3A). In a subset of 2 patients, the spatial reproducibility of mean AF voltage across a longer time period of 20 mins. Figure 3B demonstrates the 2 cases where mean AF voltage mapping was performed with an intervening period of 20 mins. Remapping separated by a 20 min period precluded paired point analysis, but we performed based upon analysis of the spatial distribution of the interpolated points.

Figure 3C shows an illustrative sample of the 2 mean AF voltage maps. While the respective locations of map points (yellow dots) vary between map 1 and map 2, spatial interpolation has been set to 5 mm. Interpolated voltage-color values are projected onto vertices that are common between the 2 maps. From these 2 cases, median ∆V_AF_ per vertex was *0.1[0.04-0.2]mV.*


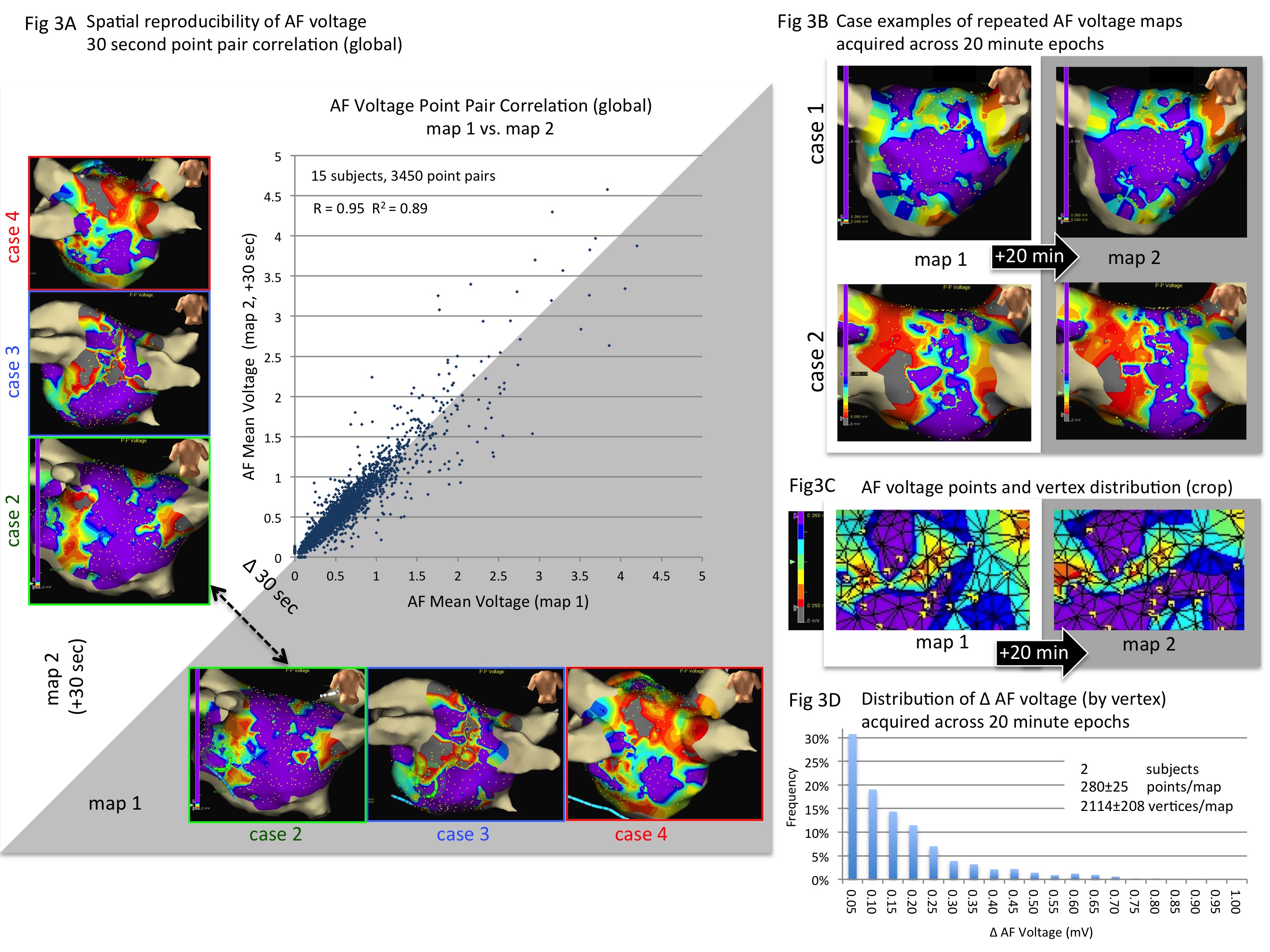


**Figure 3. Evaluating spatial reproducibility of mean AF voltage across epochs**

**(Fig. 3A)** Global correlation (15 patients, 3,450 locations) of V_mAF_ point-pairs acquired 30s apart. Cases 2,3 and demonstrate congruency of the individual maps.

**(Fig. 3B)**Two case examples of 8s V_mAF_ maps(5mm interpolation) acquired sequentially, separated by a 20min waiting period.

**(Fig 3C)** Crop of AF voltage maps acquired over a 20 minute interval. While the respective locations of map points (yellow dots) vary between map 1 and map 2, spatial interpolation has been set to 5 mm, and interpolated voltage-color values are projected onto vertices that are common between the 2 maps.

**(Fig 3D)** Distribution of the difference in AF voltage between map 1 and map 2 (Fig 3B) on a per vertex basis.  Analysis of 2114±208 vertices across the LA surface resulted in a median ∆V_AF_ per vertex of *0.1[0.04-0.2]mV.*

**References**

1. Malcolme-Lawes LC, Juli C, Karim R, et al. Automated analysis of atrial late gadolinium enhancement imaging that correlates with endocardial voltage and clinical outcomes: a 2-center study. Heart Rhythm 2013;10:1184–1191.

2. Yushkevich PA, Piven J, Hazlett HC, et al. User-guided 3D active contour segmentation of anatomical structures: significantly improved efficiency and reliability. Neuroimage 2006;31:1116–1128.
